# The common limitations in auditory temporal processing for Mandarin Chinese and Japanese

**DOI:** 10.1038/s41598-022-06925-x

**Published:** 2022-02-22

**Authors:** Hikaru Eguchi, Kazuo Ueda, Gerard B. Remijn, Yoshitaka Nakajima, Hiroshige Takeichi

**Affiliations:** 1 Human Science Course, Graduate School of Design, Kyushu University, 4-9-1 Shiobaru, Minami-ku, Fukuoka, 815-8540 Japan; 2 Department of Human Science, Faculty of Design/Research Center for Applied Perceptual Science/Research and Development Center for Five-Sense Devices, Kyushu University, 4-9-1 Shiobaru, Minami-ku, Fukuoka, 815-8540 Japan; 3 Department of Human Science, Faculty of Design/Research Center for Applied Perceptual Science, Kyushu University, 4-9-1 Shiobaru, Minami-ku, Fukuoka, 815-8540 Japan; 4 Sound Corporation, 4-10-30-103, Tonoharu, Higashiku, Fukuoka, 813-0001 Japan; 5 Open Systems Information Science Team, Advanced Data Science Project (ADSP), RIKEN Information R&D and Strategy Headquarters (R-IH), RIKEN, 1-7-22 Suehiro-cho, Tsurumi-ku, Yokohama, Kanagawa 230-0045 Japan

**Keywords:** Psychology, Human behaviour

## Abstract

The present investigation focused on how temporal degradation affected intelligibility in two types of languages, i.e., a tonal language (Mandarin Chinese) and a non-tonal language (Japanese). The temporal resolution of common daily-life sentences spoken by native speakers was systematically degraded with mosaicking (mosaicising), in which the power of original speech in each of regularly spaced time-frequency unit was averaged and temporal fine structure was removed. The results showed very similar patterns of variations in intelligibility for these two languages over a wide range of temporal resolution, implying that temporal degradation crucially affected speech cues other than tonal cues in degraded speech without temporal fine structure. Specifically, the intelligibility of both languages maintained a ceiling up to about the 40-ms segment duration, then the performance gradually declined with increasing segment duration, and reached a floor at about the 150-ms segment duration or longer. The same limitations for the ceiling performance up to 40 ms appeared for the other method of degradation, i.e., local time-reversal, implying that a common temporal processing mechanism was related to the limitations. The general tendency fitted to a dual time-window model of speech processing, in which a short (~ 20–30 ms) and a long (~ 200 ms) time-window run in parallel.

## Introduction

The effect of temporal degradation on speech perception has been investigated using several techniques, e.g., periodic interruption^1–7^, temporal smearing^8,9^, desynchronising narrow-band slits^10,11^, and temporal reversal^12,13^. Local time-reversal realises manipulation of intelligibility from perfect to almost none, depending on segment duration^7,11,14–26^. In locally time-reversed speech, an original speech waveform is divided into short segments, which are then each reversed in time, and connected again. Ueda et al.^19^ showed that, when the speech rates of individual speakers were normalised on the time axis, a segment duration shorter than about 45 ms gave almost perfect intelligibility. Intelligibility drastically decreased, however, as the segment duration increased, reaching almost floor performance for segments longer than about 100 ms. The four languages (American English, German, Japanese, and Mandarin Chinese) examined in Ueda et al.^19^ showed very similar tendencies as to the effect of segment duration on intelligibility, when the speech rates were normalised for their own data.

**Figure 1:**
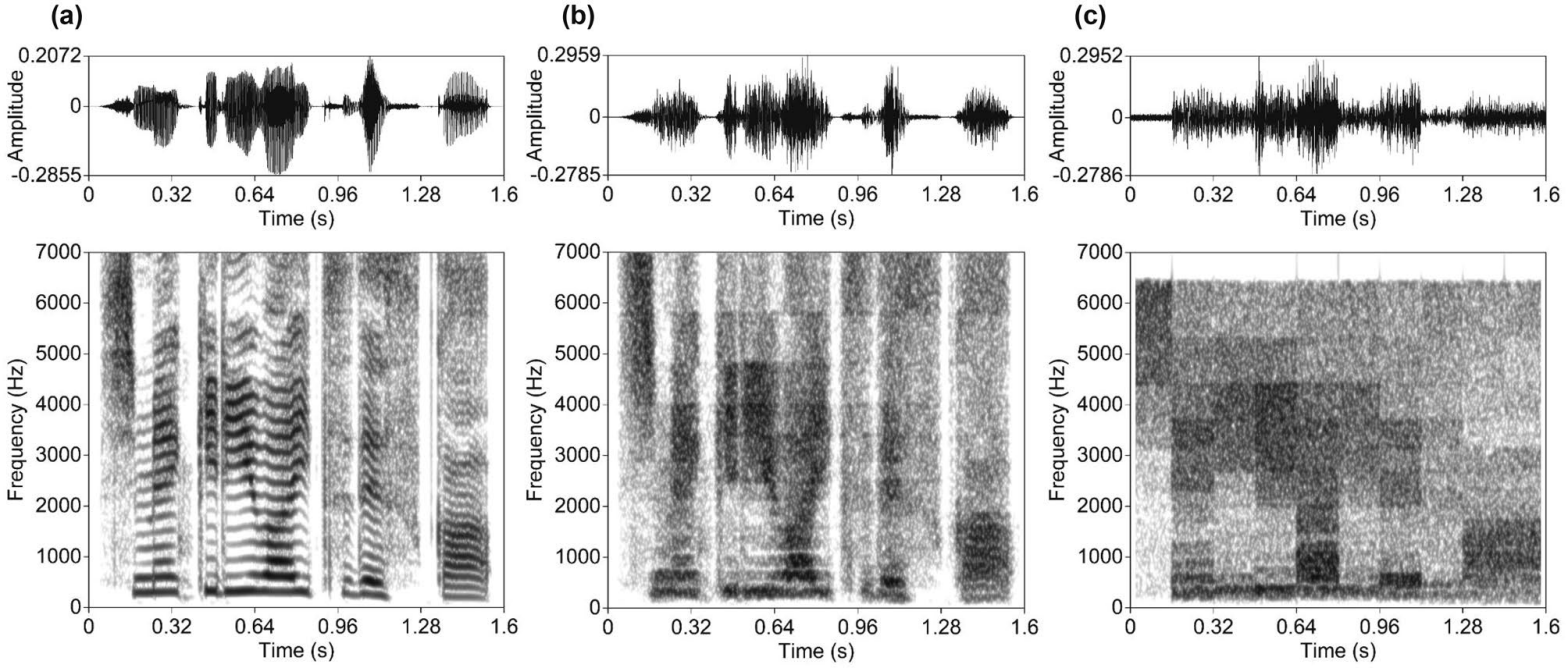
Examples of waveforms (top) and narrowband spectrograms (bottom) of the Japanese stimuli, produced from the same fragment of an original spoken sentence by a female speaker. (**a**) Original speech, (**b**) noise-vocoded speech, and (**c**) mosaic speech with 160-ms segment duration.

**Figure 2:**
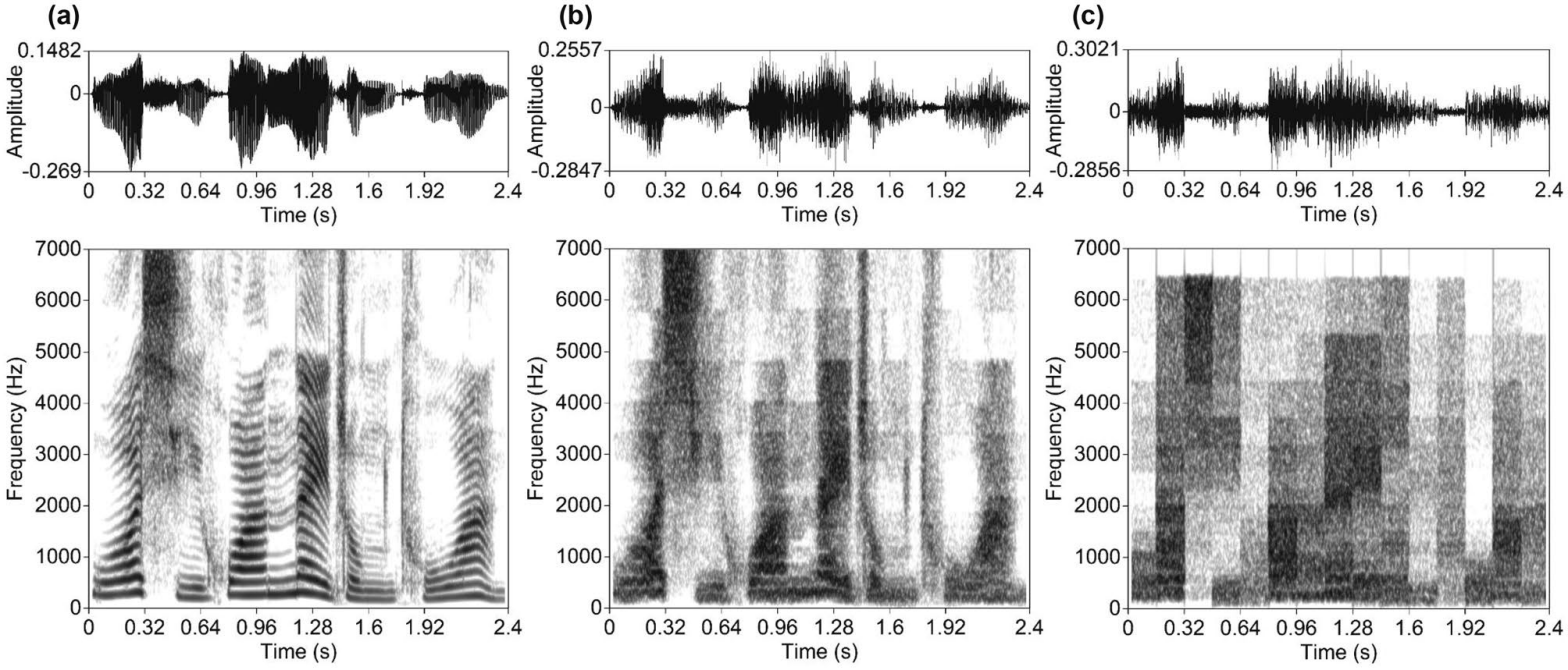
Examples of waveforms (top) and narrowband spectrograms (bottom) of the Mandarin Chinese stimuli, produced from the same fragment of an original spoken sentence by a female speaker. (**a**) Original speech, (**b**) noise-vocoded speech, and (**c**) mosaic speech with 160-ms segment duration.

On the other hand, Nakajima et al.^27^ pointed out that local time reversal not only degrades temporal resolution but also introduces misleading spectrotemporal cues, which could boost the deterioration in intelligibility. For example, reversing a segment of a rising formant transition will turn it into a falling transition, with all the implications that has for the acoustic–phonetic information being carried. This aspect of local time reversal becomes particularly prominent for longer segment durations, and leads to a sharply deteriorated intelligibility. Especially tonal languages, like Mandarin Chinese, could be subject to this. To avoid this problem, Nakajima et al.^27^ invented mosaic speech (see Figs. 1c, 2c).

Mosaic speech is closely related to noise-vocoded speech^28^ (see Figs. 1b, 2b), in which the periodicity and temporal fine structure of an original speech sample are removed and frequency resolution is degraded. In addition, in mosaic speech, temporal resolution is also degraded (cf., pointillistic speech^29^ and pixelated speech^30^). To create mosaic speech, an original speech sample is divided into small patches on a time-frequency plane and the power within each patch is averaged. Frequency resolution is determined on a critical-bandwidth scale. The resulting stepwise function in each frequency band consists of temporal segments with cosine-shaped rise and fall times of 5 ms. The amplitude envelopes are then driven by band noises of the corresponding frequency bands. Thus, in mosaic speech, periodicity and harmonic structure are lost; nevertheless, mosaic speech is still intelligible, provided that the degradation in time and frequency is moderate^27^.

In normal speech, the periodicity and temporal fine structure^31^ should be the most prominent perceptual cues for perceiving tones in a language. On the other hand, it has been proved that native listeners of Mandarin Chinese can perceive tones even in noise-vocoded speech. Specifically, the effects of frequency resolution and envelope smearing in noise-vocoded speech in Mandarin Chinese have been well-investigated^32–34^. These studies show that the listeners can perceive tones without the periodicity and temporal fine structure, provided that envelope cues are available with enough frequency resolution; nevertheless, the effects of degrading temporal resolution of amplitude envelope cues on the intelligibility of tonal languages with normalised speech rates have not yet been fully investigated. Thus, the main focus of the current investigation was to examine how degrading the temporal resolution of mosaic speech would affect the intelligibility of Mandarin Chinese (a tonal language) and Japanese (a non-tonal language), in common daily-life sentences, which were used in Ueda et al.^19^ The intelligibility was measured for the two languages with the experimental paradigm in which the segment duration was systematically manipulated and sentences from the same speech database were randomly allotted to experimental conditions across participants. The stimuli were presented to participants through the same apparatus in the same experimental environment. Care was taken to match instructions and scoring criteria in the two languages as far as possible. The results for the two languages and the previous results were examined with normalised speech rates.

## Results

**Figure 3:**
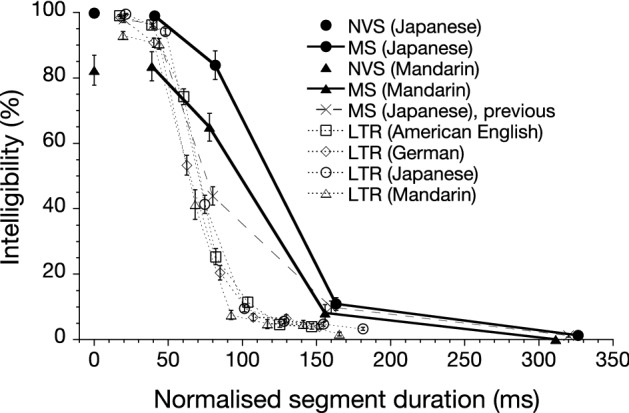
Percentage of mora or syllable intelligibility of noise-vocoded speech (NVS) and mosaic speech (MS) in Japanese ($n = 16$) and Mandarin Chinese ($n = 15$) as a function of normalised segment duration. Intelligibility of NVS is represented at 0-ms segment duration. The previous results for MS ($n = 20$) obtained by Nakajima et al.^27^  and locally time-reversed speech (LTR; $n = 28$ for American English, German, and Japanese, and $n = 27$ for Mandarin Chinese) by Ueda et al.^19^  are shown together with the present results for comparison. The segment duration is normalised with speech rates, except for Nakajima et al.^27^ Error bars indicate standard errors.

Figure 3 shows the results. The horizontal axis, the segment duration, was normalised with speech rates of the speakers, by calculating the ratio between each speaker’s speech rate and the average speech rate of the 10 speakers talking the same sentences in each language. For comparison, the mora accuracy of Japanese mosaic speech obtained by Nakajima et al.^27^, and the mora and syllable accuracy of locally time-reversed speech in four languages (American English, German, Japanese, and Mandarin Chinese) obtained by Ueda et al.^19^ are included in Fig. 3.

By and large, the present results for mosaic speech in Japanese and Mandarin Chinese showed very similar tendencies: The intelligibility decreased gradually from 99 to 11% for Japanese and from 84 to 8% for Mandarin Chinese as the segment duration increased in the 40–160-ms range. Less prominent (18% at most) were the differences between the two languages in the 40–80-ms range. The performance for noise-vocoded speech stimuli (the control condition) is designated as the 0-ms segment duration in Fig. 3. The performance difference between Japanese (100%) and Mandarin Chinese (82%) was 18% for these noise-vocoded speech stimuli. The size of the difference was quite similar to the size of the difference between the two languages for the mosaic-speech stimuli at 40-ms segment duration (15%). The speech rate differences among speakers in the two languages—as appeared in the small shifts in data points shown as black markers along the horizontal axis in Fig. 3—were negligible.

The binomial results for each mora or syllable (correct or incorrect) were analysed using Generalised Linear Mixed Models (GLMM) with a logistic linking function as implemented in an add-in for JMP Pro^35^. The data at the segmentation duration of nominal 0 (the data for the noise-vocoded speech stimuli) were excluded in the following analyses. The data were analysed for fixed effects of Language (categorical predictor), Normalised Segment Duration (continuous predictor), and an interaction of these two, and for a random effect of Participant. A variation of the model with two random effects, i.e., Participant and Sentence, failed to converge because there were so many sentences that were used just once throughout the experiment. Therefore, the model with one random effect was the only reasonable model that was applicable to the data. All statistical effects had a *p* level smaller than 0.001, unless reported otherwise. The corrected Akaike’s information criterion (AICc) was 1883.4. The analysis resulted in main effect of Language ($F(1,46.41)=18.12$) and Normalised Segment Duration ($F(1,591.3) = 131.57$), and their interaction ($F(1,591.3)= 1.27$, $p = 0.261$).

To check the validity of the analysis, the same data were analysed using a multiple beta-binomial regression^36^ model in JMP Pro^35^ for the same fixed effects as predictors. As an estimate of effect size, the area under the curve (AUC) is reported. The full model ($\mathrm {AICc}=1727.2$) resulted in a large effect size ($\mathrm {AUC}=0.95$; main effect of Language, $\mathrm {Wald}\,\chi ^2(1) = 55.45$; main effect of Normalised Segment Duration, $\mathrm {Wald}\,\chi ^2(1) = 225.22$; Language $\times$ Normalised Segment Duration interaction effect, $\mathrm {Wald}\,\chi ^2(1) = 0.88, p = 0.348$). Therefore, with both analyses, the two main effects were supported, and the interaction effect was rejected.

For noise-vocoded speech stimuli, the main effect of Language was obvious with either GLMM ($\mathrm {AICc}=65.5$; $F(1,122.5)=35.42$) or beta-binomial regression ($\mathrm {AICc}=301.8$; $\mathrm {AUC}=0.84$; $\mathrm {Wald}\,\chi ^2(1) = 41.67$).

Comparing the current results in Japanese with those from previous studies, intelligibility of nearly 100% was maintained for noise-vocoded speech, and for locally time-reversed speech or mosaic speech with shorter than 40-ms segment duration. Whereas, intelligibility declined more sharply for the locally time-reversed speech stimuli than for the mosaic speech stimuli, when the segment duration exceeded 40 ms until performance reached the floor. There was a notable difference observed between the present results and the results of Nakajima et al.^27^ at 80-ms segment duration (more than 30%), which will be discussed later.

The data obtained by Ueda et al.^19^ for locally time-reversed speech stimuli in Japanese and Mandarin Chinese ($n = 28$ for Japanese and $n = 27$ for Mandarin Chinese; shown in Fig. 3) were combined with the current data for mosaic speech stimuli and analysed with GLMM for fixed effects of Language (categorical predictor), Normalised Segment Duration (continuous predictor), Stimulus Type (categorical predictor), and all interactions. The model included Participant as a random factor. The AICc was 3001.6. The analysis resulted in main effects of Language ($F(1,103.4)=12.69$), Normalised Segment Duration ($F(1,1043)=258.30$), and Stimulus Type ($F(1,103.4)=87.05$), and interaction effects of Language $\times$ Stimulus Type ($F(1,103.4)=11.65$) and Normalised Segment Duration $\times$ Stimulus Type ($F(1,1043)=23.86$). By contrast, high *p* levels resulted in the other interaction effects, i.e., Language $\times$ Normalised Segment Duration ($F(1,1043)=1.56$, $p=0.212$) and Language $\times$ Normalised Segment Duration $\times$ Stimulus Type ($F(1,1043)=0.02$, $p = 0.875$).

The same data were submitted to the analysis with a multiple beta-binomial regression model for the same fixed effects as predictors. The full model ($\mathrm {AICc}=3421.4$) resulted in a large effect size ($\mathrm {AUC}=0.94$; main effect of Language, $\mathrm {Wald}\,\chi ^2(1) = 85.17$; main effect of Normalised Segment Duration, $\mathrm {Wald}\,\chi ^2(1) = 270.12$; main effect of Stimulus Type, $\mathrm {Wald}\,\chi ^2(1) = 244.97$; Normalised Segment Duration $\times$ Stimulus Type interaction effect, $\mathrm {Wald}\,\chi ^2(1) = 57.59$; Language $\times$ Stimulus Type interaction effect, $\mathrm {Wald}\,\chi ^2(1) = 13.38$; Language $\times$ Normalised Segment Duration interaction effect, $\mathrm {Wald}\,\chi ^2(1) = 0.82$, $p = 0.365$; Language $\times$ Normalised Segment Duration $\times$ Stimulus Type interaction effect, $\mathrm {Wald}\,\chi ^2(1) = 0.55$, $p = 0.459$). Again, both analyses supported the same statistical conclusions.

## Discussion

In sum, the main results of the current experiment are the following three. First, segment duration was the dominant factor determining the performance for both languages, Mandarin Chinese and Japanese. Second, the stimuli in both languages showed similar patterns of intelligibility variation against segment duration, that is, the ceiling performance up to 40 ms, gradual decrease thereafter, and floor performance at around 150–300 ms were observed. Third, Mandarin Chinese showed lower (15–18%) intelligibility than Japanese for both the noise-vocoded speech and the mosaic speech at 40- and 80-ms segment duration. Moreover, in a cross-study comparison, the mosaic speech stimuli showed higher intelligibility than the locally time-reversed stimuli, except for those with a ceiling performance (with segment durations shorter than 40 ms). All noise-vocoded speech stimuli and mosaic speech stimuli had a frequency resolution that was comparable to the frequency resolution of the auditory periphery, i.e., the resolution determined by critical bandwidths. Given this condition, temporal resolution was the primary factor determining the intelligibility of mosaic speech for both Mandarin Chinese and Japanese.

**Figure 4:**
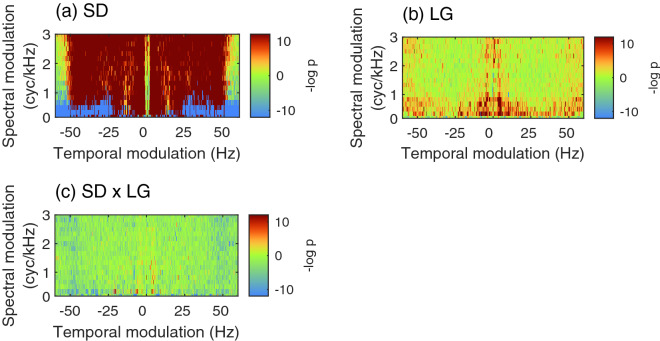
The *p*-value maps that reflect the results of two-way mixed analysis of variance (ANOVA) based on the modulation power spectra (MPS) of the stimuli. (**a**) The main effect of segment duration (SD), (**b**) the main effect of language (LG), and (**c**) the interaction effect. Hot colours indicate spectrotemporal locations at which the MPS were statistically more prominent for shorter SD and Mandarin Chinese in LG. Cold colours represent the reversed relationships. For interactions, a positive correlation between the effects is coloured hot, and a negative correlation is coloured cold.

To ensure that the mosaic speech stimuli we constructed were free of any confounding variables which may be related to the interaction effect between segment duration and language, the modulation power spectra (MPS)^37–41^ of the stimuli were calculated, according to the method provided by Flinker et al.^41^, followed by an analysis of variance (ANOVA) on the main effects of segment duration and language, and their interaction. The results showed that the main effect of segment duration was overwhelming, whereas the main effect of language was much less prominent, and that the interaction effect between these was negligible (Fig. 4). Therefore, it can be concluded that no confounding variable was included in the process of mosaicking (“mosaicising,” according to the nomenclature of Nakajima et al.^27^).

The effects of temporal degradation on intelligibility in Mandarin Chinese and Japanese thus are very similar, despite the large differences between the two languages. Among the differences, the contrast in the tones would be the most prominent one to (potentially) appear in the results. Because the stimulus manipulations that were employed in the current study were noise-vocoding and mosaicking, the periodicity and temporal fine structures in the original speech were deteriorated, apart from the manipulation of segment duration. According to the classification proposed by Rosen^31^, periodicity (50–500 Hz) provides features mainly related to the linguistic contrasts in voicing and prosody (stress and intonation), whereas temporal fine structures (600–10,000 Hz) mainly provide features for the contrasts in places of articulations and voice quality. Therefore, in Mandarin Chinese, the tone perception should be affected also by the deterioration. If the tone perception was affected, the effect may be reflected in the written-down results only for Mandarin Chinese. However, any other possible features are reflected in the results in the same way for both languages. Thus, the small discrepancy in intelligibility between the two languages can be attributed to the deteriorated perception of the tones, due to the lack of the periodicity (and temporal fine structure) in noise-vocoded speech and mosaic speech. In addition, the methods employed for taking participants’ responses may be another source of discrepancy, as we will discuss below.

The similarity calls for an explanation based on a biological mechanism that underlies temporal aspects of speech perception. Poeppel et al.^42,43^ proposed a dual time-window model of speech perception, in which a short time-window of $\sim$ 20–30 ms (for segmental units) and a long time-window of $\sim$ 200 ms (for syllable-size units) work in parallel. These time-window lengths correspond to the rhythms of neuronal oscillations in auditory cortex, the gamma rhythm and the theta rhythm. Following this model, speech segmented in units of about 30 ms (or less) should be perfectly intelligible, regardless of language. Figure 3 indeed shows that when segmented in larger units of over 40 ms, the intelligibility of both languages started to decrease. Chait et al.^43^ argued that the dual time-window model was supported by their experimental results, in which the slow ($\sim$ 4 Hz, corresponding to 250 ms) and rapid ($\sim$ 33 Hz, corresponding to 30 ms) modulations were desynchronised and no impact on intelligibility was found when delays were less than $\sim$ 45 ms. Our results are in agreement with the model with the comparable precision. The shallower slopes of the current intelligibility curves obtained with mosaic speech agree better with the length of the long time-window than the previous results with locally time-reversed speech, which reached the floor around the 100-ms segment duration (as was also discussed by Nakajima et al.^27^).

The intelligibility of Mandarin Chinese was 15–18% lower than the intelligibility of Japanese for the noise-vocoded speech and the mosaic speech with the 40-ms segment duration. There are two possible sources of differences: One is the lack of the periodicity and temporal fine structure in noise-vocoded speech and mosaic speech, and the other one is the different notation systems for responses in collecting the data from the two groups of participants. Specifically, we asked our Japanese participants to respond with hiragana or katakana, which is of daily use for the native listeners, while we asked our Chinese participants to respond with pinyin, which is not of their daily use. Therefore, the Chinese participants might have been more susceptible to making errors in their responses than the Japanese participants. On the other hand, it was difficult for us to estimate the size of increased errors with the Chinese participants; nevertheless, the proportion of errors caused with this reason can be regarded as constant over the range of segment duration. Thus, we will focus on the global effects of segment duration on intelligibility for both languages and the lack of periodicity and temporal fine structure hereafter.

The difference observed between the two languages is comparable to the difference in tone identification accuracy caused by the lack of periodicity and temporal fine structure in noise-vocoded speech^32,33^. For example, Kong and Zeng^33^ showed that lowering the cut-off frequency of the lowpass filtering for the envelopes from 500 to 50 Hz caused 20% reduction in percent correct for their 8-band noise vocoded speech in Mandarin Chinese. The present results imply that the same reason caused the lower intelligibility in Mandarin Chinese for the mosaic speech with 40-ms segment duration, because the intensity envelopes of both noise-vocoded speech and mosaic speech in our study were processed with a moving average with a Gaussian window of $\sigma = 5$ ms, which was equivalent to lowpass filtering with a cut-off frequency of 45 Hz. A closer examination of error responses that were made by the participants for Mandarin Chinese revealed that tone errors amounted to 15% for the noise-vocoded speech stimuli and 11% for the mosaic speech stimuli with 40-ms segment duration. It is to be noted that lexical tones were perceived correctly in most cases in such conditions, given that pitch information must have been limited.

It was confirmed that mosaic speech showed higher intelligibility than locally time-reversed speech except for the ceiling (at segment duration shorter than 40 ms) and floor performance (at segment duration longer than 150 ms). The comparison between the current results and the results of Ueda et al.^19^ can be made on a solid basis, because both experiments were performed with comparable procedures. The apparent discrepancy observed at 80-ms segment duration between the current results and the previous measurement for mosaic speech obtained by Nakajima et al.^27^ should be attributed to a difference in the experimental methods between the two experiments: Each stimulus was presented for three times in succession in a trial in the current experiment as well as in the experiment by Ueda et al.^19^, while each stimulus was presented just once in Nakajima et al.^27^ At the same time, the difference should not be considered major, because the general tendency of the two experimental results agreed very well, regarding the range of durations corresponding to ceiling and floor performance.

## Methods

### Participants

Sixteen native listeners of Japanese, 3 females and 13 males (age, 21–25 years; median, 22.5 years), and 15 native listeners of Mandarin Chinese, 9 females and 6 males (age, 22–30 years; median, 25 years), participated. All the participants passed a hearing test with the audiometer (RION AA-56 (RION, Tokyo, Japan)) to ensure that they had normal hearing within the frequency range of 250–8000 Hz. The research was conducted with prior approval of the Ethics Committee of Kyushu University; all methods employed were in accordance with the guidelines provided by the Japanese Psychological Association (JPA). Informed consent was obtained from all participants.

### Stimuli

A total of 200 sentences in Japanese and 78 sentences in Mandarin Chinese spoken by a female speaker of each language were extracted from the NTT-AT Multilingual Speech Database 2002 (NTT-AT, Kawasaki, Japan; recorded with a 16,000 Hz sampling rate and 16-bit linear quantisation); irrelevant blanks and noises were eliminated. The speech database utilised the sentences in articles published in newspapers and magazines in each language. Therefore, the level of complexity of the materials was the one that native speakers of each language encounter in their daily lives.

The female speaker in each language was exactly the same speaker used in Ueda et al.^19^ In Ueda et al.^19^, both a female and a male speaker were employed for the stimuli, however, the differences resulted in intelligibility between the two speakers in either Japanese or Mandarin Chinese were negligible. Our preliminary results for the current investigation ($n = 7$ for the Japanese female speaker, $n = 8$ for the Japanese male speaker, $n = 5$ for the Mandarin Chinese female speaker, and $n = 5$ for the Mandarin Chinese male speaker), employing exactly the same speakers as in Ueda et al.^19^, also showed that the differences in the obtained intelligibility scores between the female and the male speaker were negligible. Thus, we used just the samples uttered by the female speaker in each language.

Each sentence was converted into computer-oriented audio (.wav) files with a sampling frequency of 44,100 Hz using Praat^44^. Four steps of segment duration, 40, 80, 160, and 320 ms, including 5-ms cosine ramps to fade in and out, were used to make mosaic speech. In addition, noise-vocoded speech stimuli without temporal segmentation were constructed, and their segment duration was denoted nominally as 0 ms.

The speech signals were passed through a critical-band filter bank with 20 frequency bands ranging from 50 to 6400 Hz^45^. Sound energy density at each sample point in each frequency band was calculated, using a moving average of intensity with a Gaussian window of $\sigma$ = 5 ms, which was equivalent to lowpass filtering with a 45-Hz cut-off. Sound energy density was segmented according to the segment durations, and then averaged in each segment to vocode each mosaic speech stimulus. White noise was generated and divided into the same 20 critical-bands. Sound energy density of a band noise at each sample point was calculated with the same moving-averaging procedure as described above. Average sound energy density for this noise was calculated for the same segment durations. Based on the mosaicked energy density of the speech and the smoothed energy density of the noise in each frequency band, the noise extracted in the frequency band was amplitude-modulated to make the same mosaicked energy density.

In the case of noise-vocoded speech, the same procedure was applied up to the stage of calculating sound energy density at each sample point, and band-noise preparation. Following this, each band of noise was amplitude-modulated along with the obtained sound energy density without mosaicking. All stimuli were generated with an in-house program coded with the J language^46^. Figures 1 and 2 show examples of stimulus waveforms and spectrograms.

### Apparatus

The experiment was run with a computer (HP, Probook 650 G3 (HP Japan Inc., Tokyo, Japan)), using an in-house program coded with the LiveCode Community 9.0 package^47^. The stimuli were presented to the participant in a double-walled soundproof booth (Music cabin, SD3 (Takahashi Kensetsu, Kawasaki, Japan)), through a headphone amplifier with a built-in D/A converter (TEAC, UD-H01 (TEAC Corporation, Tokyo, Japan)) and headphones (Beyerdynamic, DT 990 PRO (Beyerdynamic GmbH, Heilbronn, Germany)). The sound pressure levels of the stimuli at the headphones were adjusted to 73 dB SPL, using a 1-kHz calibration tone provided with the speech database. The sound pressure levels were measured with an artificial ear (Brüel & Kjær, type 4153 (Brüel & Kjær Sound & Vibration Measurement A/S, Nærum, Denmark)), a condenser microphone (Brüel & Kjær, type 4192), and a sound level meter (Brüel & Kjær, type 2260).

### Procedure

A sequence of 30 trials for each participant consisted of six blocks of five trials/sentences. Thus, 30 combinations of the sentences and segment duration were randomly selected for each participant from 1000 possible combinations in Japanese and 390 in Mandarin Chinese. In each block, the five segmentation-duration conditions were randomly presented. The first block for each participant was treated as a set of practice trials and the results were discarded. In Japanese, 19 morae (SD $= 3.1$) per trial/sentence were presented in the experimental sessions (a mora is a syllable-like unit in Japanese). In Mandarin Chinese, 10 syllables (SD $= 1.3$) per trial/sentence were presented.

Each sentence was presented in just a single trial to each participant. The stimuli were presented diotically to the participants through the headphones. Within each trial, the sentence was repeated three times with an inter-stimulus-interval of 1 s before a participant started to answer. The participants were instructed to write down what they heard without guessing; they were asked to put what they immediately recognised, and not to fill blanks afterwards from the context. Japanese participants, who were assigned to the Japanese speech conditions, were instructed to respond in hiragana or katakana, whereas Mandarin Chinese participants, who were assigned to the Mandarin Chinese speech conditions, responded in Pinyin with tone signs. When only parts of a sentence were understood, participants described the parts at their approximate locations on a scale representing the length of a sentence. Each mora in Japanese or each syllable in Mandarin Chinese was examined as correct or incorrect, and the number of correct morae or syllables for each sentence was counted. A blank response was counted as incorrect. Homophone errors were permitted. A syllable in Mandarin Chinese was judged to be incorrect if a tone sign was incorrect. Percentage of correct morae or syllables was calculated for summarising and displaying the data, whereas statistical analysis was performed on the binomial (correct or incorrect) results.

## Data Availability

The datasets generated during and/or analysed during the current study are available from the corresponding author on reasonable request.
